# Viscoelastic properties of bovine knee joint articular cartilage: dependency on thickness and loading frequency

**DOI:** 10.1186/1471-2474-15-205

**Published:** 2014-06-14

**Authors:** Daniel M Espino, Duncan ET Shepherd, David WL Hukins

**Affiliations:** 1 School of Mechanical Engineering, University of Birmingham, Birmingham B15 2TT, UK

**Keywords:** Articular cartilage, Dynamic mechanical analysis, Stiffness, Thickness, Viscoelasticity

## Abstract

**Background:**

The knee is an incongruent joint predisposed to developing osteoarthritis, with certain regions being more at risk of cartilage degeneration even in non-osteoarthrosed joints.

At present it is unknown if knee regions prone to cartilage degeneration have similar storage and/or loss stiffness, and frequency-dependent trends, to other knee joint cartilage. The aim of this study was to determine the range of frequency-dependent, viscoelastic stiffness of articular cartilage across the bovine knee joint. Such changes were determined at frequencies associated with normal and rapid heel-strike rise times.

**Methods:**

Cartilage on bone, obtained from bovine knee joints, was tested using dynamic mechanical analysis (DMA). DMA was performed at a range of frequencies between 1 and 88 Hz (i.e. relevant to normal and rapid heel-strike rise times). Viscoelastic stiffness of cartilage from the tibial plateau, femoral condyles and patellar groove were compared.

**Results:**

For all samples the storage stiffness increased, but the loss stiffness remained constant, with frequency. They were also dependent on cartilage thickness. Both the loss stiffness and the storage stiffness decreased with cartilage thickness. Femoral condyles had the thinnest cartilage but had the highest storage and loss stiffness. Tibial plateau cartilage not covered by the meniscus had the thickest cartilage and lowest storage and loss stiffness.

**Conclusion:**

Differences in regional thickness of knee joint cartilage correspond to altered frequency-dependent, viscoelastic stiffness.

## Background

In this study, Dynamic Mechanical Analysis (DMA) has been used to determine viscoelastic stiffness of bovine knee joint articular cartilage. A frequency sweep was used to measure viscoelastic stiffness from normal up to rapid loading rates. Measurements were taken from distinct knee joint regions. This has provided a range of viscoelastic stiffness for healthy knee joints over normal and rapid loading frequencies.

Rapid heel-strike rise times during gait have been implicated in the early onset of osteoarthritis (OA) [1,2]. Heel-strike rise time is typically 100 to 150 ms [3]. A subset of the population with heel-strike rise times from 5 to 25 ms have been identified as being predisposed to OA [2]. The timing of these heel-strikes correspond to loading frequencies of 3–5 Hz for normal and up to 90 Hz for rapid heel-strike rise times [4].

Increased loading frequency alone might predispose cartilage to becoming damaged. This was suggested because of changes in cartilage viscoelastic properties with frequency such that, at higher frequencies, more energy was available to damage cartilage [4]. This damage is distinct from that caused purely by excess loading [5,6]. Undamaged articular cartilage contributes to smooth joint motion, aided by a surface roughness of around 80 – 170 nm [7]. However, OA is associated with damaged cartilage and impaired/painful joint motion [8].

Viscoelastic properties of a material are characterised by its storage and loss moduli [9,10]. The storage modulus characterises the ability to store energy which is then available for elastic recoil. The loss modulus characterises the ability of the material to dissipate energy. Storage and loss moduli are calculated from the storage and loss stiffness, respectively, using a shape factor [4]. If the area of cartilage loaded is constant (e.g. constant indenter area), the only shape factor variable is the cartilage thickness.

The mechanical properties of cartilage vary across the knee [11] and may be a result of diverse localised loading [12]. However, it is unknown how storage and loss stiffness of cartilage vary across the knee and whether all knee joint cartilage follows the frequency-dependent viscoelastic trends found by Fulcher *et al*. [4] from normal up to rapid loading. Therefore, it is also unknown if regions across the knee known to show signs of cartilage damage have a similar storage and/or loss stiffness to regions that show no signs of damage. There are two clear reasons why the knee is of particular interest. Firstly, it is an incongruent joint predisposed to developing OA [12,13]. Secondly, it often shows signs of damage at specific regions even in otherwise healthy joints [14].

The aim of this study was to determine the range of frequency dependent viscoelastic stiffness of articular cartilage across the knee joint. Bovine cartilage was used because it is an accepted model for human cartilage, with similar thickness [15]. DMA was used to measure storage and loss stiffness, with cartilage thickness subsequently measured. The ratio of storage to loss stiffness, i.e. propensity to failure, was also derived. Variation of these parameters was assessed against both frequency of loading and cartilage thickness.

## Methods

### Specimens

Six bovine knee joints, approximately between 18 and 30 months old, were obtained from a supplier (Johnston’s Butcher, Kings Heath, Birmingham, UK). Upon arrival in the laboratory, samples were dissected into femoral condyles, patellar groove and medial and lateral tibial plateau. Individual samples were then wrapped in tissue paper saturated in Ringer’s solution and stored at −40°C in plastic bags. Prior to testing, samples were thawed and individual test specimens were obtained. Such freeze-thaw treatment does not alter the dynamic mechanical properties of cartilage [16]. Test specimens included the underlying subchondral bone which prevents tissue swelling [17]; further details are available elsewhere [4,18]. Typically over 1 cm of underlying bone was maintained. India ink (Loxley Art Materials, Sheffield, UK) was used to identify any pre-existing surface lesions [19]. Regions showing pre-existing lesions were not tested. However, as joints were not osteoarthritic, large scale damage was not evident. Therefore, it was always feasible to identify a region of interest for testing. In some cases, however, this meant testing near a lesion site. Specimens were dissected so as to load an approximately flat cartilage surface, described further elsewhere [4].

### DMA frequency sweep

The experimental protocol has been defined previously [4]. Briefly, samples were secured in a custom made rig with acrylic polymer cement (WHW Plastics, Hull, UK) bathed in Ringer’s solution at room temperature. WinTest DMA software (Bose Corporation, ElectroForce Systems Group, Minnesota, USA) was used to control a materials testing machine (Bose ElectroForce 3200). A sinusoidally varying compressive force between 16 N and 36 N was applied, i.e. nominal peak stress of 1.7 MPa, estimated physiological for cartilage [20]. Forces were applied using a cylindrical indenter (diameter of 5.2 mm), with a chamfered end to prevent cartilage damage at the edge of the contact area. The cartilage surface (>20 mm × 20 mm) was larger than the indenter surface area. The resulting displacements were consistent with our previous studies [4,18]. The loading was applied over a range of frequencies (1, 8, 10, 12, 29, 49, 71, and 88 Hz). Two preload conditions were applied before the frequency sweep, at 25 and 50 Hz (1500 and 3000 cycles respectively, i.e. 2 minutes of preconditioning cycles with a 60 s rest period). Note, that the DMA protocol involved a dynamic load amplitude which resulted in a dynamic displacement amplitude that was used to calculate dynamic stiffness. This requires the cartilage to have achieved a dynamic “steady-state”, which for *ex vivo* cartilage occurs after around 1200 to 4500 preconditioning cycles [4,21,22]. Further explanation, and applicability, of the method is provided elsewhere [4,18]. Samples were fully immersed in Ringer’s solution during tests.

The cartilage thickness was measured after the final test using a previously described technique [4,23]. Briefly, a sharp needle is pushed through the cartilage layer and up to the bone using a testing machine. This is an established technique used to measure *ex vivo* cartilage thickness [15,23]; extended discussion on its applicability is provided elsewhere [18]. A total of 99 DMA frequency sweep tests were performed on the samples obtained (see *specimens* section, above).

### Calculations

The storage (*k’*) and loss (*k”*) stiffness and thickness were obtained. As the indenter diameter (5.2 mm) is constant, the only variables are the measured sample thickness and dynamic stiffness. A curve was fitted to the graphs of storage stiffness against frequency of the form:

(1) $$k'=A\log_{e}\left(f\right)+B$$

where *A* defines the gradient of *k’* plotted against the natural logarithm of *f*, the loading frequency (Hz), and *B* is the intercept. A similar curve fit has been used for representing storage modulus previously [4].

The ratio of storage to loss stiffness (*k’*/*k”* ratio) is a measure of energy stored in a system that enables its elastic recoil to the energy dissipated by the system. The greater the *k’*/*k”* ratio, the greater the proportion of energy available in the system for recoil and/or failure.

Further explanation of dynamic stiffness is available elsewhere, including its relation to dynamic modulus and phase angle [4,10,18,24].

### Statistics

Statistical comparisons of data were made between cartilage test samples from different regions within the knee joint. Cartilage test samples were identified as being either femoral or tibial. Femoral cartilage was further classified as being either from the femoral condyles or the patellar groove. Tibial plateau cartilage was classified as either meniscus covered or not covered by the meniscus. Medial and lateral samples were compared to determine whether medial-lateral differences were negligible, thereby, enabling results to be combined. A one-way analysis of variance (ANOVA) was undertaken using Tukey’s method for multiple comparisons to investigate significant differences (p < 0.05), in thickness and/or dynamic mechanical stiffness and moduli, between the different regions [25]. Tables in the results section use ^*A, B, C, D, E*^ to indicate ‘statistical’ groups which are significantly different. Also, a knee joint region may form part of more than one ‘statistical’ group. Thus, where two knee joint regions do not share a letter they are significantly different (p < 0.05) but if they do share a letter they are not significantly different (p > 0.05) from each other.

Frequency dependent measurements of storage and loss stiffness, and their ratio, were compared at all frequencies. For conciseness, comparisons at 1 Hz are presented in the results. Note, the two constants, *A* and *B*, are used to characterise the frequency dependent storage stiffness of samples (equation 1); i.e. their comparisons are not made at an individual frequency. Dynamic moduli were compared across different knee regions to assess whether regional variations were due to inherently different material properties of cartilage.

Cartilage thickness was compared across knee joint cartilage. Lines of best fit between cartilage thickness and storage and loss stiffness, and their ratio, were plotted to determine whether correlations were significant (p < 0.05). Regression equations were obtained for significant correlations.

## Results

### Lateral and medial cartilage

At 1 Hz the only significant difference in viscoelastic stiffness between comparable lateral and medial cartilage was limited to the tibial plateau. The lateral tibial plateau cartilage, that is covered by the meniscus, had a significantly greater loss stiffness than the corresponding medial cartilage (Table 1). For subsequent analyses, data obtained from lateral and medial samples were combined.

**Table 1:** Mean and standard deviation of parameters measured at 1 Hz and thickness of cartilage

		***n***	**Thickness (mm)**		**k’ (N/mm)**		**k” (N/mm)**		**k’/k”**	
			**mean**	**SD**	**mean**	**SD**	**mean**	**SD**	**mean**	**SD**
Lateral	Femoral condyle	12	0.89^C^	0.24	949^A^	266	95^A,B^	24	10.0^A^	1.5
Medial	Femoral condyle	12	0.89^C^	0.29	897^A^	203	118^A^	29	7.9^A^	1.8
Lateral	Patellar groove	9	1.48^B,C^	0.18	508^B,C,D^	135	57^C,D,E^	20	9.3^A^	2.1
Medial	Patellar groove	8	1.14^C^	0.22	708^A,B^	235	80^B,C^	21	9.1^A^	3.0
Lateral	Tibial plateau		0.98^C^	0.43	763^A,B^	190	98^A,B^	29	8.0^A^	1.1
	meniscus covered	14								
Medial	Tibial plateau		1.22^C^	0.29	534^B,C^	197	60^C,D^	11	8.8^A^	2.7
	meniscus covered	13								
Lateral	Tibial plateau not meniscus covered	15	2.36^A^	0.5	249^D^	160	29^E^	14	8.1^A^	2
Medial	Tibial plateau not meniscus covered	16	2.03^A,B^	0.78	360^C,D^	204	45^D,E^	28	8.3^A^	1.8

The letters^A, B, C, D, E^ are used to indicate significant differences between the different regions; where a region does not share a letter they are significantly different (p < 0.05).

*n:*number of samples tested from each region.

### Thickness

From the samples tested, cartilage thickness ranged from 0.5 to 3.3 mm. Tibial plateau cartilage not covered by the meniscus was significantly thicker than all other knee joint cartilage (2.19 ± 0.67 mm, p < 0.05; Table 2). In descending order, it was followed by patellar groove and meniscus covered tibial plateau cartilage. The thinnest cartilage was found at the femoral condyles (0.89 ± 0.26 mm; Table 2).

**Table 2:** **Storage (*k’ *) and loss (*k”*) stiffness, and their ratio, at 1 Hz and cartilage thickness**

	***n***	**Thickness (mm)**		**k’ (N/mm)**		**k” (N/mm)**		**k’/k”**	
		**mean**	**SD**	**mean**	**SD**	**mean**	**SD**	**mean**	**SD**
Femoral condyles	24	0.89^A^	0.26	923^A^	233	106^A^	28	9.0^A^	2.0
Patellar groove	17	1.32^B^	0.26	614^B^	215	69^B^	23	9.2^A^	2.5
Tibial plateau meniscus covered	31	1.10^A,B^	0.38	653^B^	223	80^B^	29	8.4^A^	2.0
Tibial plateau not meniscus covered	27	2.19^C^	0.67	304^C^	189	37^C^	23	8.2^A^	1.8

The letters^A, B, C^ are used to indicate significant differences between the different regions; where a region does not share a letter they are significantly different (p < 0.05).

*n:*number of samples tested from each region.

### Loss stiffness

**Figure 1:**
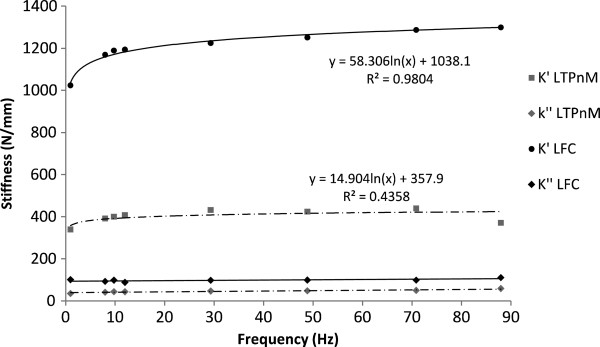
**Frequency dependency of storage (*k’*) and loss (*k”*) stiffness for two cartilage samples.** The two samples shown were from the lateral tibial plateau, not under the meniscus (LTPnM), and lateral femoral condyle (LFC). The former was 2.1 mm and the latter 0.9 mm thick. A trend-line, of the form of equation 1, is included for the storage stiffness of both samples. Note, in the equations in the graph *x* refers to the frequency (*f* in equation 1) and *y* to the storage stiffness (*k’* in equation 1).

The loss stiffness of articular cartilage (on bone) was frequency independent (Figure 1). The loss stiffness of knee joint cartilage ranged from 16 – 640 N/mm. Femoral condyle cartilage has a significantly greater loss stiffness than all other knee joint cartilage (106 ± 28 N/mm, p < 0.05; Table 2). It was followed by patellar groove and meniscus-covered tibial plateau cartilage. Tibial plateau cartilage not covered by the meniscus had a significantly lower loss stiffness than all other cartilage (37 ± 23 N/mm, p < 0.05; Table 2).

**Figure 2:**
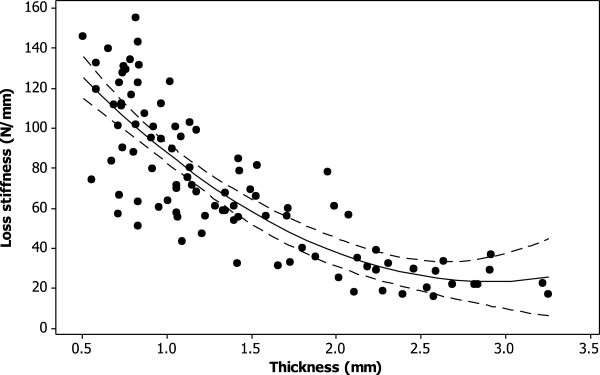
**Thickness dependent loss stiffness of knee joint cartilage at 1 Hz.** A second order polynomial curve (see equation 2) was fitted to the experimental data (*R*^*2*^ = 69%; p < 0.05) as shown (solid line). The 95% confidence intervals are also included (dashed line).

There was a significant non-linear decrease in the loss stiffness with thickness across knee joint cartilage (p < 0.05; Figure 2). A second order polynomial curve fitted to the experimental data (equation 2; *R*^*2*^ = 69%) was

(2) $$k''=172.8-102.7t+17.64t^{2}$$

where *t* is the cartilage thickness in mm, and *k”* is the loss stiffness in N/mm.

### Storage stiffness

**Figure 3:**
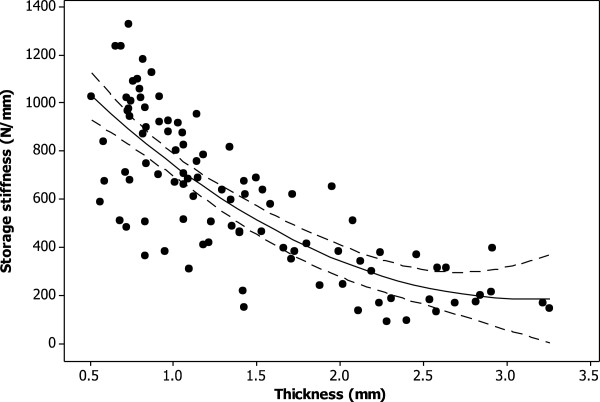
**Thickness dependent storage stiffness of knee joint cartilage at 1 Hz.** A second order polynomial curve (see equation 3) was fitted to the experimental data (*R*^*2*^ = 63%; p < 0.05) as shown (solid line). The 95% confidence intervals are also included (dashed line).

The storage stiffness ranged from 90 to 1930 N/mm. At 1 Hz, tibial plateau cartilage not covered by the meniscus, had a significantly lower storage stiffness than all other cartilage (304 ± 189, p < 0.05; Table 2). Femoral condyle cartilage had significantly higher storage stiffness values than all other cartilage (923 ± 233, p < 0.05; Table 2; Figure 1). The storage stiffness decreased significantly with thickness (p < 0.05; Figure 3), with data being fitted using a second order polynomial equation (equation 3; R^2^ = 63%).

(3) $$k'=1384-764.1t+121.7t^{2}$$

where *k’* is the storage stiffness in N/mm.

**Figure 4:**
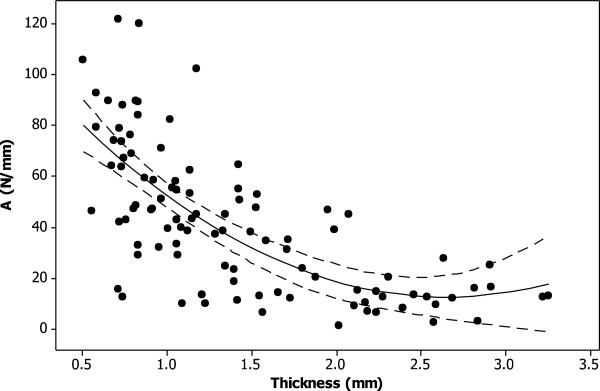
**Thickness dependency of *A *(see equation 1) for knee joint cartilage.** A second order polynomial curve (see equation 4) was fitted to the experimental data (*R*^*2*^ = 51%; p < 0.05) as shown (solid line). The 95% confidence intervals are also included (dashed line).

For all samples storage stiffness increased with frequency (Figure 1). This relationship was described using two constants, *A* and *B* (see *Calculations* section). Constant *A* ranged from 2 to 120 N/mm. Tibial plateau cartilage not covered by the meniscus, was significantly lower than all other cartilage (21 ± 18, p < 0.05; Table 3). Femoral condyle cartilage had significantly higher values for *A* than all other cartilage (67 ± 29, p < 0.05; Table 3). Constant *A* decreased significantly with thickness (p < 0.05), this relationship can be described using a second order polynomial equation (equation 4; R^2^ = 51%; Figure 4).

**Table 3:** Variation in storage stiffness coefficients with knee joint location

	**A (N/mm)**		**B (N/mm)**	
	**mean**	**SD**	**mean**	**SD**
Femoral condyles	67^A^	29	922^A^	231
Patellar groove	38^B^	24	584^B^	225
Tibial plateau meniscus covered	45^B^	21	656^B^	223
Tibial plateau not meniscus covered	21^C^	18	303^C^	182

The letters^A, B, C^ are used to indicate significant differences between the different regions; where a region does not share a letter they are significantly different (p < 0.05).

(4) $$A=115.5-77.81t+14.68t^{2}$$

**Figure 5:**
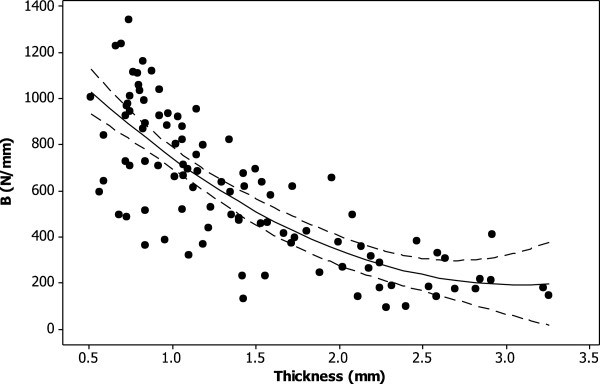
**Thickness dependency of *B *(see equation 1) for knee joint cartilage.** A second order polynomial curve (equation 5) was fitted to the experimental data (*R*^*2*^ = 63%; p < 0.05) as shown (solid line). The 95% confidence intervals are also included (dashed line).

The intercept *B* ranged from 90 to 1340 N/mm. Tibial plateau cartilage not covered by the meniscus had significantly lower *B* values than all other cartilage (303 ± 182, p < 0.05; Table 3). Femoral condyle cartilage had significantly higher values for *A* than all other cartilage (922 ± 231, p < 0.05; Table 3). Constant *B* decreased significantly with thickness (p < 0.05), with data being fitted using a second order polynomial equation (equation 5; R^2^ = 63%, Figure 5).

(5) $$B=1393-781.2t+126.9t^{2}$$

### Ratio of k’/k”

From the samples tested, the *k’/k”* ratio (i.e. ratio of storage to loss stiffness ratio) for cartilage ranged from 1–14. This ratio increased with frequency as *k’*, but not *k”*, increased with frequency (see *Loss* and *Storage stiffness* sections, above). No significant differences in the *k’/k”* ratio were determined for different cartilage samples (p > 0.05; Table 2). For example, tibial plateau cartilage not covered by the meniscus had the lowest value for the *k’/k”* ratio (8.2 ± 1.8). However, its *k’/k”* ratio was not significantly lower than the *k’/k”* ratio for patellar groove cartilage (9.2 ± 2.5) which had the highest ratio.

### Dynamic moduli

Tibial plateau cartilage, not covered by the meniscus, had significantly lower storage and loss moduli than the thinnest knee joint cartilage of the femoral condyles (Tables 4 and 5). These findings are consistent with findings for dynamic stiffness (see *storage stiffness* and *loss stiffness*, above).

**Table 4:** **Storage (*E’ *) modulus, at 1 Hz and 90 Hz and cartilage thickness**

	***n***	**Thickness (mm)**		**1 Hz (E’, N/mm**^**2**^**)**		**90 Hz (E’, N/mm**^**2**^**)**	
		**mean**	**SD**	**mean**	**SD**	**mean**	**SD**
Femoral condyles	24	0.89^A^	0.26	37.3^A^	8.2	52.3^A^	9.2
Patellar groove	17	1.32^B^	0.26	35.9^A,B^	9.5	44.4^A,B^	12.9
Tibial plateau meniscus covered	31	1.10^A,B^	0.38	31.7^A,B^	11.2	40.9^B,C^	13.6
Tibial plateau not meniscus covered	27	2.19^C^	0.67	27.6^B^	12.0	32.3^C^	16.2

The letters^A, B, C^ are used to indicate significant differences between the different regions; where a region does not share a letter they are significantly different (p < 0.05).

*n:*number of samples tested from each region.

**Table 5:** **Loss (*E” *) modulus at 1 Hz and cartilage thickness**

	***n***	**Thickness (mm)**		**E” (N/mm**^**2**^**)**	
		**mean**	**SD**	**mean**	**SD**
Femoral condyles	24	0.89^A^	0.26	4.3^A^	1.0
Patellar groove	17	1.32^B^	0.26	4.0^A,B^	1.1
Tibial plateau meniscus covered	31	1.10^A,B^	0.38	3.8^A,B^	1.1
Tibial plateau not meniscus covered	27	2.19^C^	0.67	3.3^B^	1.2

The letters^A, B, C^ are used to indicate significant differences between the different regions; where a region does not share a letter they are significantly different (p < 0.05).

*n:*number of samples tested from each region.

At 1 Hz, femoral condyle cartilage had the largest storage modulus (37.3 ± 8.2 N/mm^2^; Table 2). It was followed by patellar groove and meniscus-covered tibial plateau cartilage. Tibial plateau cartilage not covered by the meniscus had a significantly lower storage modulus (27.6 ± 12 N/mm^2^) than femoral condyle cartilage (p < 0.05; Table 4). Storage moduli at 90 Hz were significantly greater than those at 1 Hz (p < 0.05), consistent with a frequency dependent increase in storage stiffness (see *storage stiffness*, above). Results at 90 Hz were consistent with findings at 1 Hz and for stiffness, with mean storage moduli ranging from 52.3 ± 9.2 N/mm^2^ (femoral condyles) to 32.3 ± 16.2 N/mm^2^ (tibial plateau cartilage not covered by the meniscus; Table 4). Thus, storage modulus of the thinnest cartilage increased with frequency by 40%, compared to a 17% increase for the thickest cartilage.

Femoral condyle cartilage had the largest loss modulus (4.3 ± 1.0 N/mm^2^; Table 5). It was followed by patellar groove and meniscus-covered tibial plateau cartilage. Tibial plateau cartilage not covered by the meniscus had a significantly lower loss modulus than femoral condyle cartilage (3.3 ± 1.2 N/mm^2^, p < 0.05; Table 5). Values are reported for 1 Hz, however, note that the loss modulus was not frequency dependent.

## Discussion

### Main findings

This study has used dynamic mechanical analysis to characterise the storage and loss stiffness (i.e. viscoelastic stiffness) of articular cartilage, on bone, across bovine knee joints. The ratio of storage to loss stiffness was also derived. The dependency on both frequency and cartilage thickness was assessed for all parameters. Key findings from this study include:

▪ femoral condyles had the thinnest cartilage but had the highest storage and loss stiffness;

▪ tibial plateau cartilage not covered by the meniscus was the thickest cartilage across the knee with the lowest storage and loss stiffness;

▪ the storage and loss stiffness were dependent on cartilage thickness;

▪ no differences in propensity to fail (i.e. *k’/k”* ratio) were found across the knee joint;

▪ changes in moduli confirm that stiffness trends result from inherent changes in the material properties of the tissue rather than simply size.

### Storage and loss stiffness

In this study we found the storage, but not loss, stiffness to be frequency dependent. These findings are consistent with previous reports of moduli between frequencies of 1 – 90 Hz for cartilage on-bone [4]. This was expected because the storage and loss moduli are calculated from the storage stiffness and a shape factor, where the shape factor is a constant for an individual sample. The loss modulus has been reported as being frequency dependent when removed from its underlying bone [9]. However, the physical behaviour of cartilage when on and off-bone differs [17] because of the restrictions provided by the underlying bone to cartilage [26]. For example, hysteresis can decrease when cartilage is on-bone, but increase when off-bone, with increased loading velocity [27].

Storage and loss stiffness both decreased with cartilage thickness. Therefore, femoral condyle cartilage and tibial plateau cartilage not covered by the meniscus appear to represent extremes across the knee. Cartilage from the patellar groove and from the tibial plateau covered by the meniscus fell somewhere in between. Moreover, both the gradient and intercept increased with reduced cartilage thickness. Therefore, thinner cartilage had a greater storage stiffness increase with frequency than thicker cartilage.

### Knee joint & implications for the onset of OA

Tibial plateau cartilage not covered by the meniscus was the thickest cartilage in our knee joints tested and was most likely to show signs of damage. Damage to this cartilage is common, and has even been reported in otherwise healthy adolescent human knee joints [14]. It has also been shown that this cartilage is thicker than other knee cartilage by weakening of its underlying structure [14]. For example, a comparison of collagen fibers of tibial plateau cartilage, showed that cartilage not covered by the meniscus were of more uniform diameter and more likely to be arranged in parallel bundles than cartilage under the meniscus [14]. Collagen provides the main tensile reinforcement in connective tissues [10,28]. Thus, changes in collagen uniformity and alignment are expected to compromise its ability to provide such reinforcement during dynamic loading. This is consistent with our finding that the cartilage not covered by the meniscus had the lowest dynamic stiffness.

It has been argued that tibial plateau cartilage not under the meniscus is exposed to intermittent shock-loading with low ambulatory stress [12]. This combination of loading has been proposed to lead to degeneration of cartilage for all joints [29,30]. Our results show that this degenerated, thicker, cartilage also has reduced dynamic stiffness and a reduced frequency-dependent increase in storage stiffness. As mentioned above, increased cartilage thickness is a consequence of damaged ultrastructure. However, tibial plateau cartilage not under the meniscus also has greater water content (87%) than meniscus covered cartilage (71%) [14]. Therefore, differences in dynamic stiffness, and its frequency dependency, are expected to be the result of changes to gel-collagen interaction. How, structurally, cartilage dissipates and stores energy is likely to be related to the stress transfer mechanism during gel-collagen interaction [31].

In the knee joint, higher than baseline bone joint area and volume of cartilage has been associated with the loss of cartilage over the subsequent two-years [32]. Antony *et al*. [32] suggested that the most likely cause of increased cartilage volume was swelling. This is consistent with increased water content and cartilage thickening during the early stages of OA [33]. Our results show that increased thickness is accompanied by reduced storage and loss stiffness. The *k’/k”* ratio had a similar range of values to a previous study [18], but did not vary with thickness. Therefore, no differences in propensity to fail are reported due to differences in storage to dissipation of energy. However, if thicker cartilage is weaker than thinner cartilage, then for a given *k’/k”* ratio it may experience greater damage. This is consistent with tibial plateau cartilage not covered by the meniscus being damaged across the knee [14].

### Relevance to heel-strike

A subset of the population with high heel-strike rise times during gait may be implicated in the onset of OA [1]. This subset of the population has been identified as having heel-strike rise times ranging from 5–25 ms [2,3,34]. These rise times are sufficiently rapid to generate impulsive loading during heel-strike. They compare to ‘healthy’ heel-strike rise times of approximately 100–150 ms [3,4].

Our experiments include frequencies of up to 92 Hz which leads to a maximum rise time of 5.4 ms, comparable to impulsive heel-strike rise times [4]. Although heel-strikes repetitively occur at this rate only after an individual gait cycle, this does provide a maximum frequency at which to measure mechanical stiffness. Moreover, it highlights how cartilage dynamic mechanical properties change with heel-strike rise times.

Experimentally, this led to an increase in storage to loss stiffness with frequency, and is in agreement with previous findings of an increased ratio of storage to loss modulus [4] and stiffness [18] with frequency. Thus, there is an increase in the proportion of energy stored to energy dissipated which is due purely to the increase in frequency. If the energy stored becomes too great, then excess energy would potentially be dissipated through cracking the tissue; i.e. cartilage failure. This mechanism would be consistent with cartilage failure occurring through increased energy during impact loading [35].

If energy storage and dissipation is altered with loading frequency, then the transfer of energy during contact from bone to cartilage and cartilage to bone could also be altered. The altered energy transfer, with loading frequency, to the underlying bone may have implications for bone remodelling.

### Limitations

In this study lateral and medial results were pooled to enable comparisons between regions. This meant that a limited number of lateral-medial differences were ignored. The only difference identified was limited to lateral tibial plateau cartilage, that is covered by the meniscus, having a significantly greater loss stiffness than the corresponding medial cartilage. However, full medial-lateral data (including statistical analysis) has been presented. The advantage of combining medial-lateral cartilage is that it enables comparison of cartilage under similar generic-types of loading. For medial-lateral comparisons, using Table 1, it should be noted that animal knee joints undergo lower external tibial rotation, during knee extension, than the human knee joint [36]. Differences in rotation may lead to subtle medial-lateral differences in tibial human cartilage not applicable to animal knee joint cartilage.

A bovine knee joint model has been used in this study. Bovine cartilage is an accepted ultrastructural model for human cartilage [37] and has been shown to be a good mechanical model for the knee joint including similar thickness [15]. While animal models are often used to infer human knee joint function [38] they remain a model for a human joint. Due to similarities in the joint similar trends across different regions of the knee are anticipated, i.e. we do not anticipate large changes to our conclusions. However, known differences in medial-lateral rotation, in particular, should be noted when comparing tibial medial and lateral behaviour and extrapolating conclusions to human cartilage.

A possible limitation with cyclic loading at higher frequencies for any soft structure is that the displacement is not able to recover its original position with load. This could lead to wave addition through the sample, cycle on cycle. This effect was not observed during our tests, consistent with previous articular cartilage studies testing cartilage up to 90 Hz [4,18] and 100 Hz [16] at the macro-scale, or up to 300 Hz at nano-scale [39].

## Conclusions

This is the first study to correlate dynamic stiffness of articular cartilage, at healthy and traumatic loading frequencies, to its underlying thickness. Viscoelastic stiffness of knee joint articular cartilage are thickness and frequency dependent. Thin knee joint cartilage (e.g. femoral condyles) had higher storage and loss stiffness; whereas, thick cartilage (e.g. cartilage from the tibial plateau not covered by the meniscus) had lower storage and loss stiffness. Changes to the storage and loss stiffness are such that the proportion of stored to dissipated energy does not change across the joint. However, thicker cartilage may be predisposed to failure because of its weaker structure; note thick knee joint cartilage is known to be structurally compromised [14].

## Competing interests

The authors declare that they have no competing interests.

## Authors’ contributions

DME participated in conception and design of the study, performed acquisition of all data, analysis, interpretation of data, and drafted the manuscript. DETS participated in conception and design of the study and has critically revised the manuscript for important intellectual content. DWLH participated in conception and design of the study and has critically revised the manuscript for important intellectual content. All authors read and approved the final manuscript.

## Pre-publication history

The pre-publication history for this paper can be accessed here:

http://www.biomedcentral.com/1471-2474/15/205/prepub
